# A novel investigation using thermal modeling and optimization of waste pyrolysis reactor using finite element analysis and response surface methodology

**DOI:** 10.1038/s41598-023-37793-8

**Published:** 2023-07-06

**Authors:** Shivi Garg, Anand Nayyar, Abdulrajak Buradi, Krushna Prasad Shadangi, Prabhakar Sharma, Bhaskor Jyoti Bora, Akshay Jain, Mohd Asif Shah

**Affiliations:** 1 Energy Institute, Bengaluru, Centre of Rajiv Gandhi Institute of Petroleum Technology, Bangalore, Karnataka 562157 India; 2 Graduate School, Faculty of Information Technology, Duy Tan University, Da Nang, 550000 Viet Nam; 3 Department of Mechanical Engineering, Nitte Meenakshi Institute of Technology, Bangalore, Karnataka 560064 India; 4 Department of Chemical Engineering, Veer Surendra Sai University of Technology, Burla, Sambalpur, Odisha 768018 India; 5 Department of Mechanical Engineering, Delhi Skill and Entrepreneurship University, New Delhi, 110089 India; 6 Department of Economics, Bakhtar University, Kabul, 2496300 Afghanistan; 7 School of Business, Woxsen University, Kamkole, Sadasivpet, Hyderabad, Telangana 502345 India; 8 Division of Research and Development, Lovely Professional University, Phagwara, Punjab 144001 India

**Keywords:** Renewable energy, Energy infrastructure

## Abstract

The influence of humans on the environment is growing drastically and is pervasive. If this trend continues for a longer time, it can cost humankind, social and economic challenges. Keeping this situation in mind, renewable energy has paved the way as our saviour. This shift will not only help in reducing pollution but will also provide immense opportunities for the youth to work. This work discusses about various waste management strategies and discusses the pyrolysis process in details. Simulations were done keeping pyrolysis as the base process and by varying parameters like feeds and reactor materials. Different feeds were chosen like Low-Density Polyethylene (LDPE), wheat straw, pinewood, and a mixture of Polystyrene (PS), Polyethylene (PE), and Polypropylene (PP). Different reactor materials were considered namely, stainless steel AISI 202, AISI 302, AISI 304, and AISI 405. AISI stands for American Iron and Steel Institute. AISI is used to signify some standard grades of alloy steel bars. Thermal stress and thermal strain values and temperature contours were obtained using simulation software called Fusion 360. These values were plotted against temperature using graphing software called Origin. It was observed that these values increased with increasing temperature. LDPE got the lowest values for stress and stainless steel AISI 304 came out to be the most feasible material for pyrolysis reactor having the ability to withstand high thermal stresses. RSM was effectively used to generate a robust prognostic model with high efficiency, R^2^ (0.9924–0.9931), and low RMSE (0.236 to 0.347). Optimization based on desirability identified the operating parameters as 354 °C temperature and LDPE feedstock. The best thermal stress and strain responses at these ideal parameters were 1719.67 MPa and 0.0095, respectively.

## Introduction

**Figure 1:**
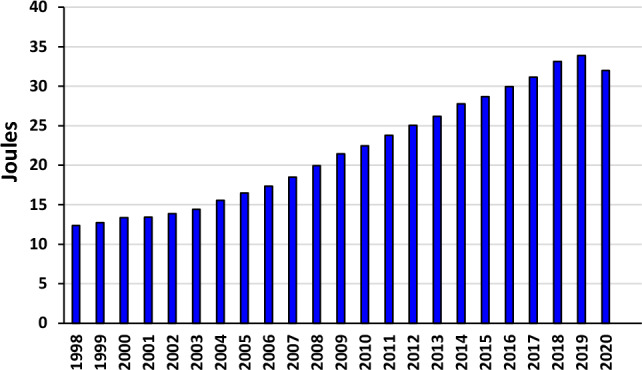
India’s energy consumption from 1998 to 2020.

In recent decades, the application of renewable energy resources is gradually increasing day by day due to the continuous shrinking of fossil fuels and their resources^1,2^. Energy plays a crucial role in any country’s economic development^3^. India has a strong influence on the global energy market^4^. In a view of the growing population of India since 2000, the energy demand has increased more than double (Fig. 1). The increasing urbanization and industrialization ensure rise in India’s energy demand. There is a huge difference in the energy use pattern across different states and between rural and urban areas. Two factors that are crucial to Indian consumers are affordability and reliability of energy sources^5,6^.

Humans have been utilizing a wide variety of biomass resources as a key renewable energy resource for the past many years due to their carbon–neutral content and play a major role in controlling the harms concerning the change of climate as per the climate change information. The advent of fossil fuels, this dependency is somewhat reduced. However, recent increasing transportation fuel demand, over-dependence on fossil fuels, increasing global warming and climate change suggests that efforts should be made to shift our economy towards the renewable sector^7,8^. For a sustainable future, a pollution-free environment, less dependency on fossil fuels and oils, clean air to breathe, and water to drink, humanity needs to move towards the usage of renewable sources available in nature^9–11^. Biomass feedstock is one such useful resource present in nature. Apart from giving a clean and sustainable environment, this has the potential of giving new opportunities to young people^12,13^. Although research is going on this topic and many technologies are already available, still there is a long way to go for the products to be economically accepted and feasible. The social perspectives suggest that a bio-based economy will not only benefit the environment but will also open doors to more employment in the farming and industrial sectors. As seen in the past few years, the renewable sector has achieved significant growth^14,15^.

In many countries, biomass resources are not sufficient to satisfy the energy demands and this situation is termed a ‘secondary energy crisis’. The problem of rising oil prices goes along with this problem^16^. There is a need to develop ways to utilize renewable energy sources from the countries to mitigate this dual-energy problem. The society must be aware of all the implications that biomass resources might cause, both good and bad. There could be many barriers to the utilization of biomass resources^17^ such as infrastructure, financial and other resources, manufacturing knowledge and technological aspects. However, the immense potential of biomass is one encouraging factor in terms of a green economy with subsidiary benefits^18,19^.

**Figure 2:**
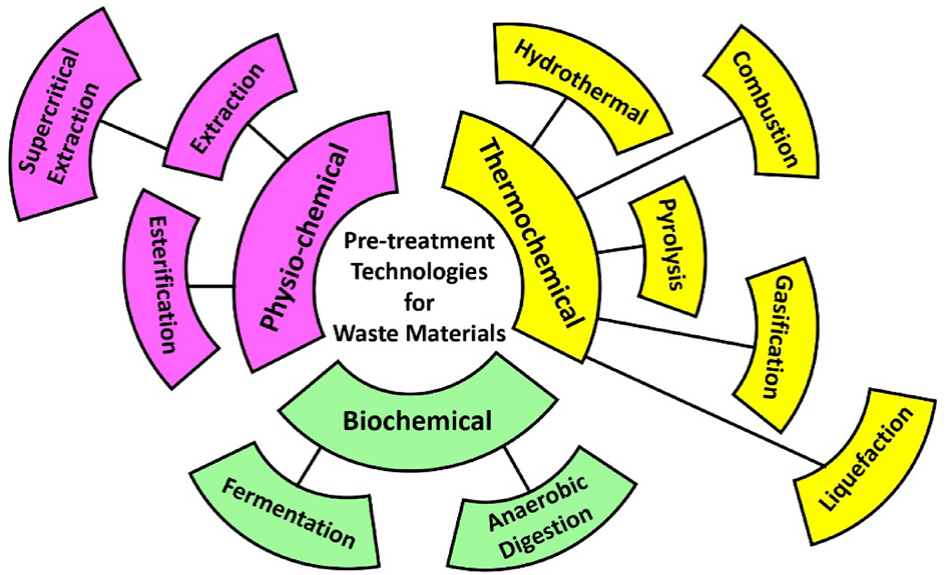
Waste material conversion technologies.

Recent era has another problem along with the energy crisis is the air pollution caused by the increased usage of plastic materials^20^. Most of the plastic waste is either ends to landfills or incinerated. These processes are harmful for the environment and causes air pollution. Increased dumping of plastics into landfills pollutes the environment which ultimately degrade the ecosystem^21^. The world is surrounded by plastics. From the syringe to the computer, everything is made of plastic. Even the Covid19 protective gear are made of plastic. Due to these applications in today's life, people have become dependent on this one-time-use article. The ways through which plastic can be eliminated are shown in Fig. 2.

Some conventional ways of waste management include open burning or incineration, which leads to pollution of air. The burning of plastic waste releases many toxins such as carbon monoxide, volatile organic compounds, smoke, and polycyclic aromatic hydrocarbons into the air^22^. One of the most advanced techniques to convert waste plastic in to fuel is pyrolysis^23^.

Pyrolysis involves the conversion of feedstock into a mixture of oils, gases, and solid charcoal. The application to heat to the feedstock in the absence of oxygen breaks it down into small chain molecules. The pyrolysis process is divided into two phases: The first phase deals with the fragmentation of molecules and depolymerization of polymers into monomers. This phase takes place within a temperature range of 150–300 °C. Free radicals are generated in this stage which leads to the formation of hydroxyl, and carboxyl groups. Free radicals contain lone pairs, which are responsible for the formation of reactive oxygen^24,25^. The second phase executed in the temperature range of 400–600 °C and it includes polymerization, cracking, and condensation. Large molecules tend to deposit on the walls after polymerization which hinders the overall process by reducing the efficiency. By varying different process conditions, pyrolysis oil may be obtained which acts as fuel.

The by-products which are not completely vaporized and consist fixed carbon and ash, which are referred to as char. The problem with the pyrolysis process is that the paralytic oil that we get as the product is thermally unstable due to a large amount of oxygen present in it. Hence, hydrogenation and catalytic cracking of the oil are required to make it thermally stable^26^. Another hurdle with pyrolysis is the generation of flu gas. However, if this gas further treated, then this process can work wonders for the existing problems of plastics and biomass degradation. Pyrolysis is classified based on heating rates as slow pyrolysis or (non-isothermal), in which volatile organic material evaporates partly leaving behind a large part of solid carbon as the main product, called char. Fast pyrolysis or (isothermal) is the one in which feedstock is heated at elevated temperatures within a short span to make pyrolytic oil. The reactor is considered to work isothermally to maximize the product yield. And ultra-flash/flash pyrolysis is an extremely rapid process with a high heating rate and product yield and short residence time^27^.

Thermal pyrolysis is a method of depolymerization of materials under very high temperatures in the absence of oxygen. Products include gaseous, liquid, and solid fuel. The condensable volatile fraction forms the liquid fuel, and the remaining non-condensable part is a high calorific value gas. Product properties vary significantly with the type of feedstock. In microwave-assisted pyrolysis^28^ the wavelength of microwave radiation ranges from one millimetre to one meter and its frequency ranges from 300 MHz to 30 GHz. Microwave heating is mainly used for instantaneous starting and stopping and for generating rapid heating, which results in higher yields and selectivity of the compounds. Residence time, moisture content, temperature rate, and concentration of microwave absorber are the factors that play a crucial role in determining product quality. High temperature and residence time with low heating rates favor high gas production. In the case of solar pyrolysis, the heat is provided by concentrated solar radiation^29^. Compounds crack into smaller fractions in the primary stage having a temperature range of around 250–500 °C.

In the second stage primary heavy compounds further, crack into smaller molecules at a temperature of 700 °C. In slow pyrolysis, the main product is char because of low temperatures, slow heating rates, and high residence time, while in fast pyrolysis the main product is liquid fuel owing to high temperatures, high heating rates, and low residence time. The main product of solar pyrolysis is a liquid fuel, which means it follows fast pyrolysis^30^. The most common type of reactor used here is a direct heating reactor in which there is one glass window and a concentrator in the reactor to focus the concentrated radiation on the feedstock. This method proves to be a better alternative in terms of heat and efficiency. There is one indirect heating method also, but due to high heat losses and complicated heat transfer circuits, it is not at all preferred.

### Objectives of paper

The following are the objectives of paper:

- To conduct the examination of thermal stress and thermal strain experienced by the pyrolysis reactor as a result of high temperatures and pressures. In this regard, four different materials of the reactor along with four different feedstocks are considered. The simulation study for determination thermal stress and thermal strain is carried out Fusion 360 simulation software.
- To perform modeling and optimization of thermal stress and thermal strain for different feeds and reactor materials of the pyrolysis process through Response Surface Methodology.

### Organization of paper

“Literature review” section highlights literature review that reveals previous work related to the current topic. “Materials and methods” section discusses the materials and methods used in the investigation, as well as the subsequent optimization and numerical approaches. The results are then given and discussed in “Results and discussion” section. “Conclusion and future scope” section concludes the paper with future scope.

## Literature review

Various experimental and computational investigations highlighted the effect of multiple parameters on pyrolysis. In this regard, Wang et al.^30^ tested with different pyrolysis temperatures, feed rates of rice straw and Camellia oleifera shell using microwave radiation as the heating source. The outcome of the study was reported that the yield of bio-oil increased with an increase in temperature in the case of rice straw while it decreased with an increase in temperature in the case of camellia oleifera. However, the pyrolytic oil/bio-oil that we get from thermal pyrolysis requires a high-temperature range and retention time. Also, the quality of the oil was less. These issues can be rectified by using a catalyst. Shadangi et al.^31^ studied thermal and catalytic pyrolysis of Karanja oil seeds. The experiment was performed in a semi-batch reactor and aimed at finding out the optimum temperature for pyrolysis. CaO, Kaolin, and Al_2_O_3_ were used as catalysts in different ratios with the feed. Composition analysis revealed that the pyrolytic oil produced through catalytic pyrolysis had a higher calorific value as compared to the pyrolytic oil produced from thermal pyrolysis. Das et al.^32^ analyzed the samples of LDPE, High-Density Polyethylene, and PP at a slow dynamic condition. It was reported that the pyrolysis temperature played an important role in determining the composition of the pyrolytic oil. Also, the concentration of paraffin increased with increasing temperatures while the concentration of olefin decreased. In a study reported by Barbarias et al.^33^, a mixture of PS, PE, PP, and polyethylene terephthalate was assessed for pyrolysis. The process was divided into two parts, the first being the pyrolysis step and the other reforming step. The products were analysed by gas chromatography and micro-gas chromatography. For the catalytic process to work efficiently, the perfect choice of the reactor is required. Various types of reactors have been proposed Shadangi et al.^31^ for pyrolysis in the literature for commercial use. The most common type of reactor is the fluidized bed reactor which is mainly preferred for the catalytic processes. These reactors are flexible to various feeds and have got high heat and mass transfer rates. It was concluded that high heating rates were due to the proper mixing of biomass material with the bed material. The reactor material plays an important role in handling high heat and pressure. Stainless steel is the most widely used reactor material for pyrolysis due to its corrosion-resistant properties. Moreover, it can withstand high temperatures and pressure as reported by a few studies^34,35^. The simulation study done by Sadhukhan et al.^36^ consisted of analyzing the effects of temperature and particle size. A mathematical model was formulated, and it was solved using the Finite Volume Method with Tri-Diagonal Matrix Algorithm approach. The model was further verified by the experimental results.

The thermal stress–strain behavior of the pyrolysis reactor’s body is critical, yet it is a complicated process. Its modeling with first principles is difficult and fraught with ambiguity. However, modern machine learning techniques, paired with faster computing power, give a foundation for developing parametric modeling and optimization^37^. This is a more realistic approach to modeling engineering problems and systems. It employs analysis of variance (ANOVA) on data gained via experimental research. The response surface methodology (RSM) has been used in recent times in various engineering fields such as biofuel research^38^, dual-fuel combustion^39^, emission modeling^40^, and nanofluids characterization^41^ among others. Several authors have employed RSM to optimize the operating parameters of pyrolysis reactors to optimize their output. Das and Goud^42^ employed RSM to maximize bio-oil output. A quadratic model based on ANOVA was built to analyze the trend and connection between the test responses and the process parameters, namely nitrogen mass flow rate (0.87–1.5 LPM), temperature (300–600 °C), and holding duration (20–60 min). The temperature had the greatest impact, followed by holding duration and nitrogen flow rate. In another study, Tripathi et al.^43^ employed RSM to improve the output of oil palm shell-based char and Brunauer–Emmett–Teller (BET) surface area by optimizing process parameters. The operating parameters for maximizing OPS char production (60.93%) and BET surface area (250.03 m^2^/g) were determined using the ANOVA analysis of the experimental data. The anticipated findings were confirmed, and it was discovered that the experimental data differed from the projected values by only 5.99% in yield and 6.34% in BET surface area.

The literature review indicates that there has not been enough research done on the behaviour of different feeds in terms of thermal stress and thermal strain values. Also, the effect of different reactor materials has not been touched upon yet. Another significant research gap is the non-availability of modeling-optimization studies in this domain. According to the literature review, even though RSM is a rigorous modeling-optimization approach, its potential use in the pyrolysis reactor's thermal stress–strain paradigm for modeling prediction has not been examined.

## Materials and methods

### Software analysis

**Figure 3:**
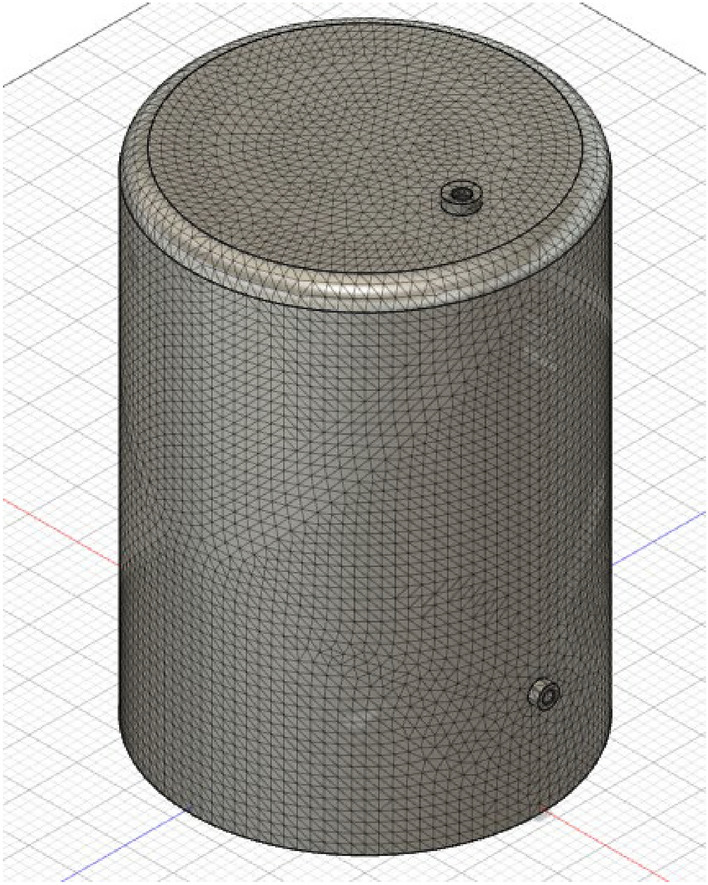
Meshing with element size of 100 mm.

**Figure 4:**
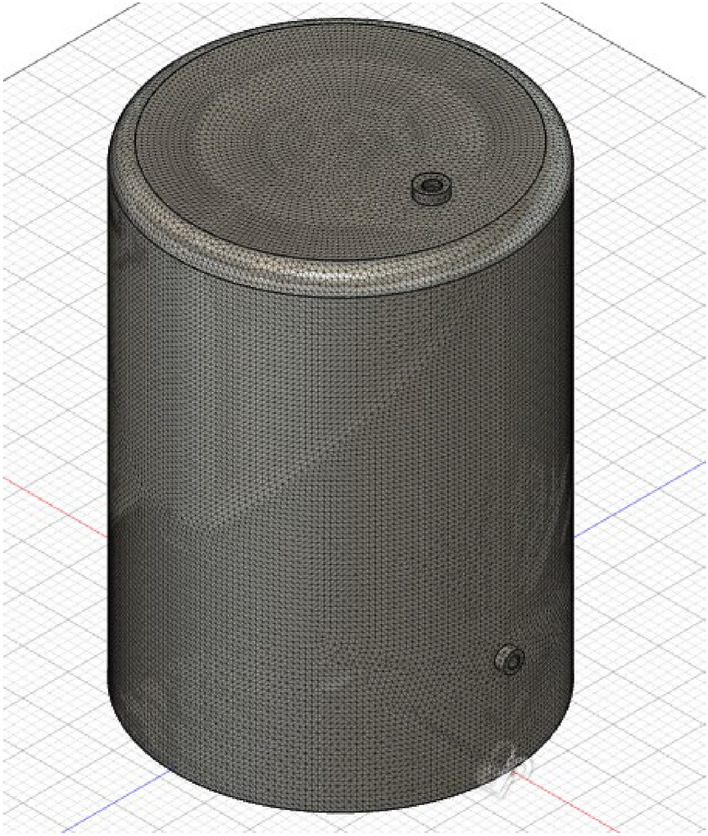
Meshing with element size of 50 mm.

**Figure 5:**
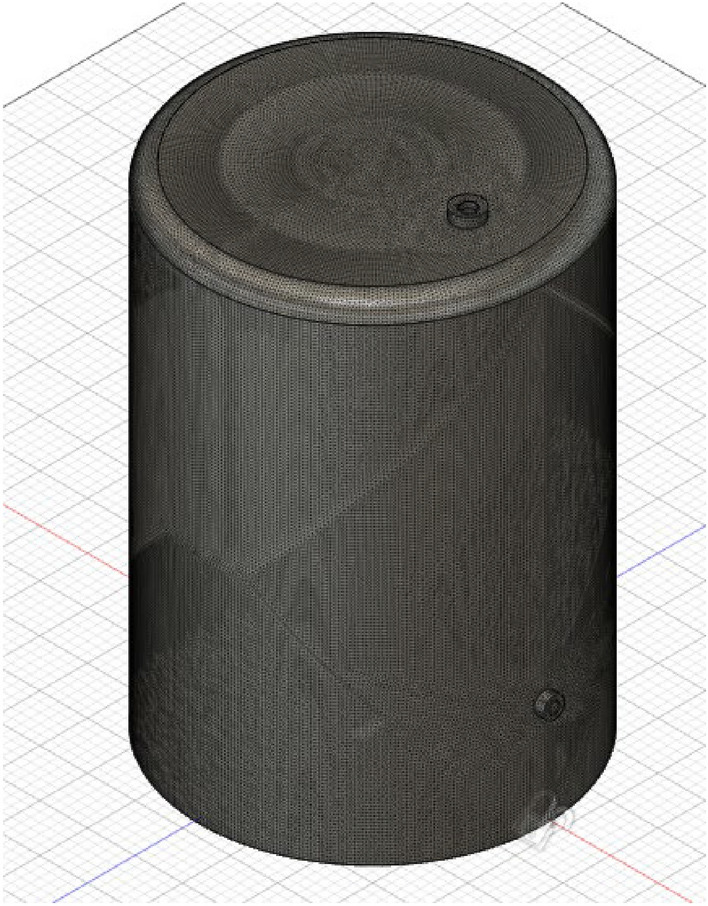
Meshing with element size of 30 mm.

**Figure 6:**
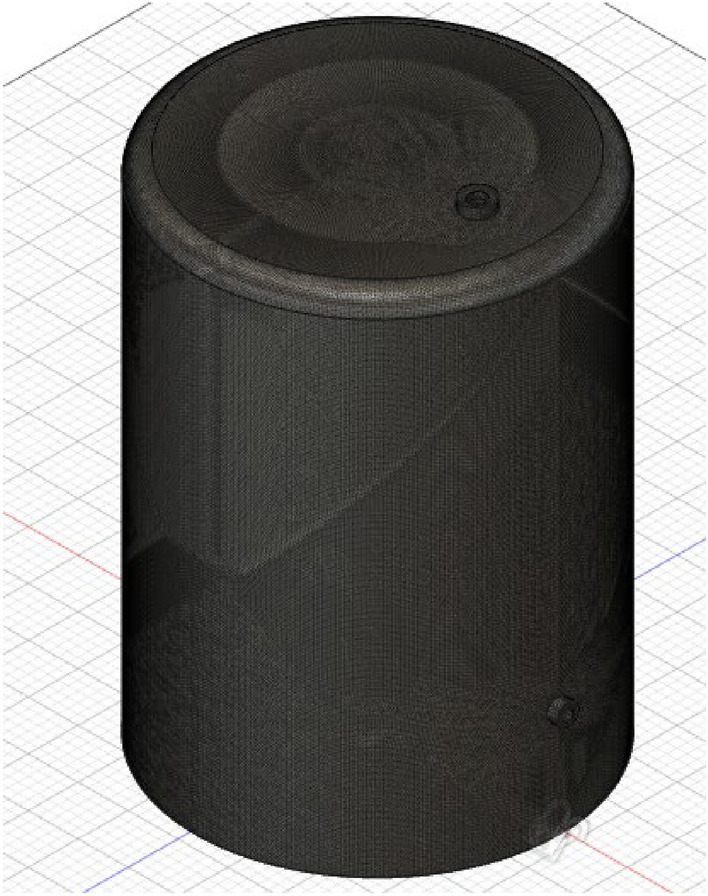
Meshing with element size of 20 mm.

Fusion 360 simulation software was used for doing a 3D analysis of the pyrolysis reactor based on different feedstock and different reactor materials. This software gives us the steady state analysis while taking into account the heat flow. Hence, atleast one heat load is required to simulate the heat flow. Also, the thermal conductance in this software takes the value infinity by default, if not stated otherwise. While doing this analysis, meshing was performed on the reactor for Finite Element Analysis (FEA). Fusion 360 works on the principle of the Finite Element approach. Table 1 shows the materials which were considered in this study. One feed was selected at a time and its thermal and structural analysis was done. The thermal analysis gave the temperature profile for that particular feed and that profile was further used to get the thermal stress and strain values. Figures 3, 4, 5 and 6 show meshing refinement which was done to get accurate results and to minimize the error. The dimension of the Pyrolyser is referred from a previous study^44^.

**Table 1:** Computational matrix.

Feeds	Reactor materials
LDPE	Stainless steel AISI 202
Wheat straw	Stainless steel AISI 302
Pinewood	Stainless steel AISI 304
PS + PE+PP	Stainless steel AISI 405

### Governing equations behind fusion 360 software

- Thermal analysis of the pyrolysis reactor is based on the Heat Transfer concepts of conduction and convection (Assumption 1).
- Conduction is used while studying the temperature profile throughout the reactor, whereas convection helps to understand the heat transfer from the reactor body to the surroundings (Assumption 2).
- Thermal analyses are steady-state heat transfer analyses used to determine the steady-state temperature distribution and heat flow (Assumption 3).
- The thermal conductivity of the material must be known as well as the ambient temperature and heat transfer coefficients at convection (Assumption 4).
- Heat is always transferred in the direction of decreasing temperature (Assumption 5).

Following equations are used to show these concepts:

1. Heat Transfer:
  1 $${\text{Q }} = {\text{ mc}}\Delta {\text{T,}}$$
  where m = mass of the system, c = Specific heat of the system, ∆T = Change in temperature of the system, Q = Heat transferred.
2. General Conduction:
  2 $$\text{Fourier's law}, ,\text{ Q }= -\text{k A }\frac{dT}{dx},$$
  where k = Thermal conductivity of the material, A = Area of the surface, $\frac{dT}{dx}$= Temperature gradient.
3. Convection:
  3 $${\text{Newton's law of cooling}}, {\text{ Q }} = {\text{ hA }}\Delta {\text{T,}}$$
  where h = Heat transfer coefficient of the material, A = Area of the surface, ∆T = Temperature difference between surface and surroundings.
  Structural analysis is explained using the concept of coefficient of thermal expansion, which results in generating stresses throughout the reactor body.
  Volumetric thermal expansion, β is used to explain the expansion of the reactor.
  The outer diameter of the reactor after the expansion can be calculated as follows:
    4 $${\text{D}}_{{1}} = {\text{ D}}_{0} \left( {{1 } + \, \beta {\text{T}}} \right),$$
    where D_1_ = diameter after expansion, D_0_ = diameter before expansion.

### Mesh independence test

In an FEA model, the geometry is divided into a series of discrete points called nodes. This process is called meshing. Too many nodes indicate more computation time and a smaller number of nodes indicate inaccurate results. Hence, to come to an accurate solution, an optimized number of nodes should be chosen so that the results are independent of the mesh size. This process is called convergence. Mesh convergence ensures accurate results. Table 2^22,42,44^ shows different pyrolysis range based on which contours were generated for other feeds as well. It was observed that the thermal stress and strain values increased with increasing temperatures.

**Table 2:** Pyrolysis range of feeds^22,42,44^.

Feeds	Pyrolysis range
LDPE	410–465
Wheat straw	20–600
Pinewood	20–470
PE+PS + PP	20–500

**Figure 7:**
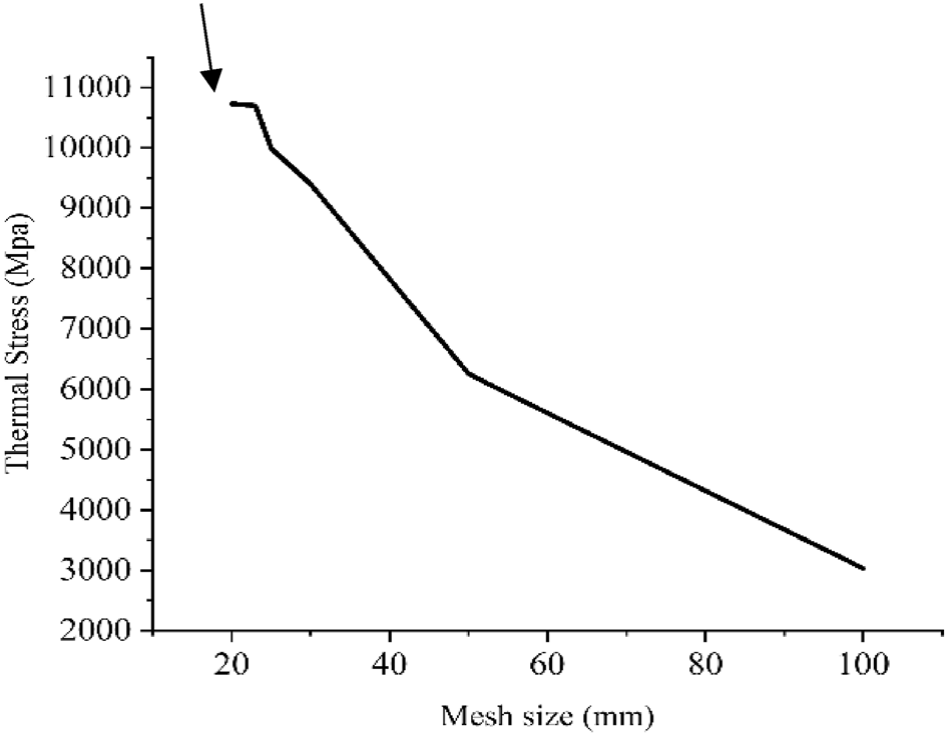
Mesh independence test.

Figure 7 depicts Mesh Independence Test which was performed to do the error analysis. The arrow in this figure points to one small horizontal line which shows that the results have reached a constant point and the value of the thermal stress is not changing with mesh size anymore.

### Model validation

**Figure 8:**
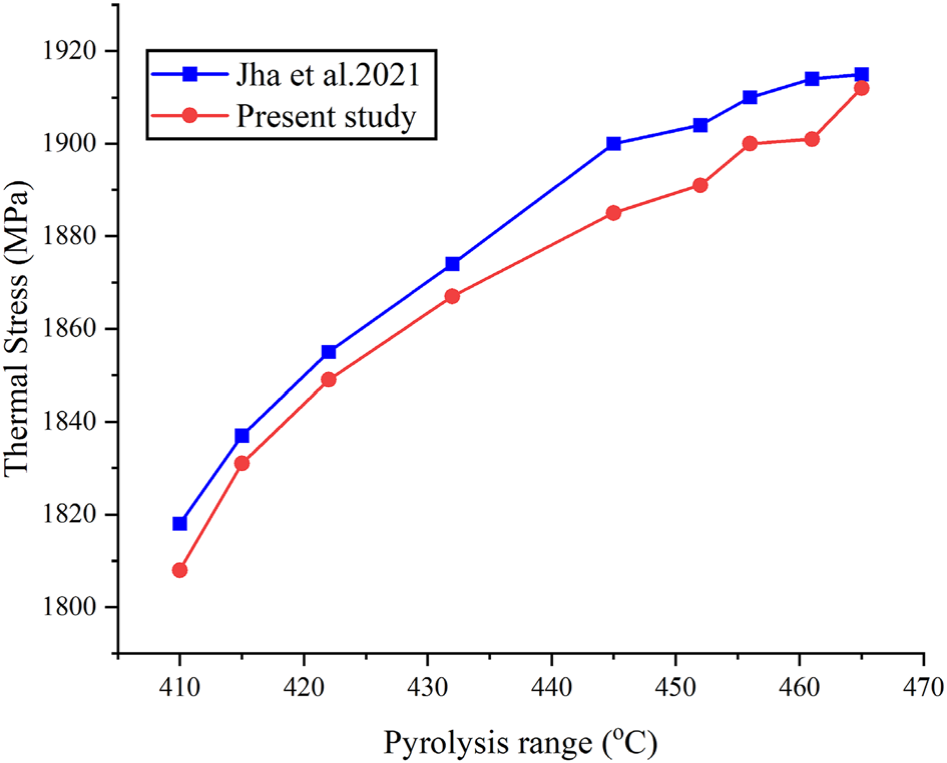
Model validation.

For the same design, same boundary conditions and turbulence models, the results obtained from the present model using Fusion 360 simulation software is compared the previous model of Jha et al.^42^ designed for biomass pyrolysis reactor. Figure 8 indicates that the present work follows a similar trend to that Jha et al.^42^ which validates the model.

### Response surface methodology

The RSM can establish the settings of the input variables and optimize a single or a series of outputs. Also, within certain constraints, RSM optimizes the optimal output responses by maximizing or decreasing process parameters. RSM was used in this work to create a predictive model that consists of a set of quadratic algebraic equations. The data collected during the experimental phase was used for the modeling. The second-degree polynomial expression denotes the RSM-based model:

5 $$y={d}_{0}+ \sum_{n=1}^{k}{d}_{i}{x}_{i} + \sum_{i=1}^{k}\sum_{j\ge i}^{k}{d}_{ij}{x}_{i}{x}_{j}+\upepsilon .$$

**Figure 9:**
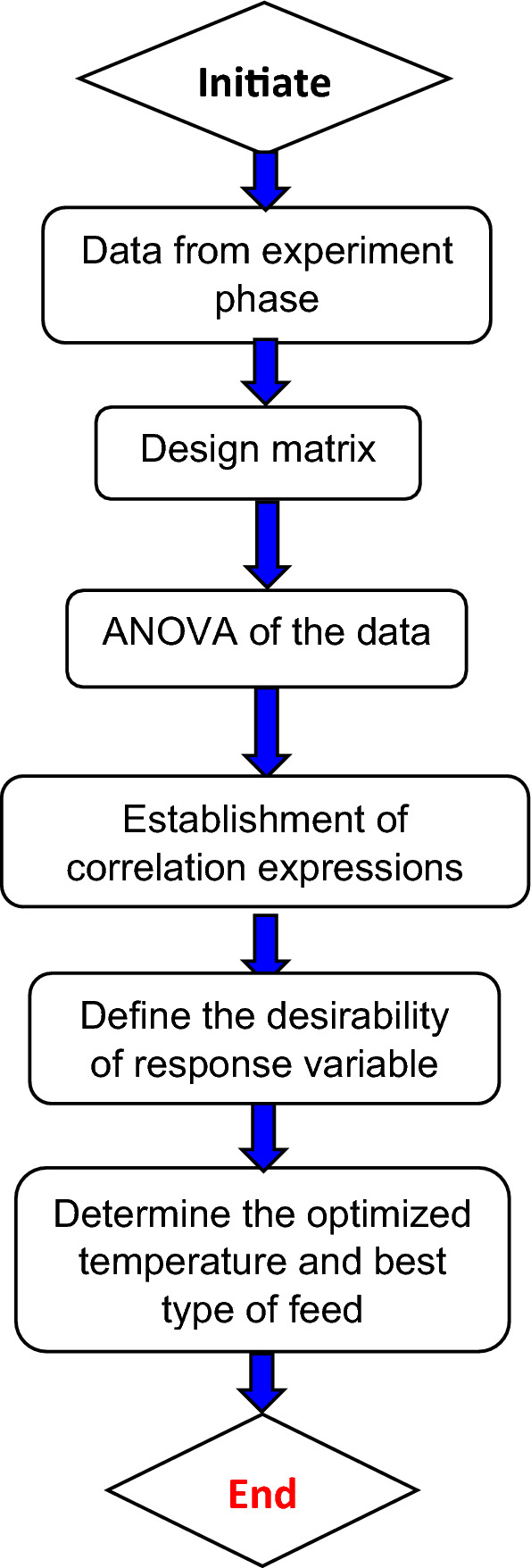
Flow chart of optimization process.

Herein, $y$ denotes the forecasted response while ${d}_{0}$ represents bias. The $i \text{ and} j$ denote coefficients for a linear and quadratic function. The ${d}_{i}$ and ${d}_{ij}$ are linear and interactive coefficients, k is the number of components, ${x}_{i}$ and ${x}_{j}$ are independent variables, and ϵ is the random error identified in the response. The RSM-based model was evaluated based on statistical measures such as coefficient of determination (R^2^), the predicted R^2^, and the adjusted R^2^. The desirability technique was utilized to do parameterized optimization to get the best outcome. The optimization investigation was carried out using Design-Expert software, which translates each response to a dimensionless desire value (), that may be obtained in the band of 0 to 1. The zero values indicate an undesirable reaction, whereas the value = 1 indicates a highly desired response. Based on the problem at hand, the objective of any solution might be to accomplish a maximum, a decrease, a target, to remain within a range, or to be equal to. The flow chart of the optimization process employed in the present study is shown in Fig. 9.

### Data collection and pre-processing

Thermal stress and strain developed during the pyrolysis of different biomass feeds and at different temperatures were modelled using ANOVA. The historical data approach was used to build the model. This software assumes that the observations are mutually independent to each other and normally distributed. These assumptions suggest that the output of one investigation should not hamper the output of the other investigation or observation. The experimental design consisted of 27 runs and 108 data points (4 parameters 27 runs). Table 3 displays the experimental research data as a designed array.

**Table 3:** Design array.

Test run	Temperature (°C)	Type of feed	Thermal stress (MPa; Von Mises)	Thermal strain (Equivalent)
1	410	LDPE	1814	0.01099
2	415	LDPE	1822	0.01112
3	422	LDPE	1834	0.0113
4	431	LDPE	1849	0.01154
5	445	LDPE	1873	0.01191
6	452	LDPE	1885	0.0121
7	458	LDPE	1895	0.01225
8	462	LDPE	1901	0.01236
9	465	LDPE	1907	0.01244
10	20	WS	0.00045	3.3E-009
11	125	WS	626.2	0.00408
12	250	WS	1372	0.00894
13	375	WS	2117	0.01381
14	500	WS	2863	0.01867
15	600	WS	3459	0.02256
16	20	PWS	0.00045	3.3E-009
17	100	PWS	477.1	0.00311
18	200	PWS	1074	0.007
19	300	PWS	1670	0.01089
20	400	PWS	2266	0.01478
21	470	PWS	2684	0.0175
22	20	PPP	0.00045	3.3E-009
23	116	PPP	572.6	0.00373
24	212	PPP	1145	0.007467
25	308	PPP	1718	0.0112
26	404	PPP	2290	0.01493
27	500	PPP	2863	0.01867

The quality of the data is quite a significant factor in the development of a strong machine learning model. The current research comprised two inputs (one categorical and one numerical) i.e., type of feed and temperature. There were two outputs namely thermal stress and thermal strain. In all, twenty-seven data sets were gathered through experimentation. Descriptive statistics were employed to highlight the nature and symmetry of the data. The findings of descriptive statistics are shown in Table 4. Descriptive statistics is also highly essential since it would be difficult to picture what the data was indicating if we just presented it as raw data. Positive skewness indicates that the probability density function’s left side is bigger than its right side. In other words, there are fewer high-value data points than low-value data points. A negative skewness value may also be seen. Table 4 shows that the majority of the output results data has a skewness of zero or very near to zero, suggesting a normal distribution. Kurtosis is a statistical concept that compares the form of the data distribution to that of the normal distribution. Kurtosis is 0 for a normal distribution, while it is negative for a flatter distribution and positive for a more peaks distribution. Values close to 0 (Table 4) suggest a normal distribution in the current circumstances. The data was subsequently submitted to outlier and nil data tests; however, no outliers or missing values were found.

**Table 4:** Descriptive statistical analysis of the data.

Particulars	Temp. (°C)	Thermal stress (MPa; Von Mises)	Thermal strain (Equation)
Mean	328.8	1628.8	0.0104
Standard error	32.52	173.47	0.0011
Median	404	1834	0.0113
Standard deviation	169.01	901.37	0.0058
Sample variance	28,566.49	812,483.8	3.43E−05
Kurtosis	− 0.794	− 0.235	− 0.209
Skewness	− 0.650	− 0.290	− 0.226
Range	580	3459	0.022
Maximum	600	3459	0.022
Minimum	20	0.00045	3.3E−09
Sum	8880	43,976.9	0.2833
Count	27	27	27

### ANOVA and development of correlation functions

ANOVA, a statistical method used to determine if the mean of two groups varied, was utilized in the present investigation. The deductive approach employs sample analysis to derive population characteristics. ANOVA assists us in determining if the sample results are relevant to populations. The p-value of the ANOVA result may be used to assess if there are statistically significant differences between some of the means. The p-value of the data was compared to a predetermined significance threshold to assess if the mean differences are statistically significant. To assess variance between groups, the F-index was estimated. A higher F-index indicates that the deviations among groups are greater than the variance within the groups. In such instances, the means of the groups are more likely to deviate.

The ANOVA results are shown in Table 5. The F-index of 3.176E+007 for the thermal stress model and 1.574E+007 for the thermal strain model indicates that it was significant enough to merit examination. It also shows that there is a 0.01% chance that such a high F-index is due to noise. The model terms are significant if the p-value is less than 0.0500. For both thermal stresses as well as thermal strain models, the model variables T, B, and TB were significant.

**Table 5:** ANOVA results.

Particulars	Thermal stress model		Thermal strain model		
	F-index	p-value	F-index	p-value	
Model	3.176E+007	< 0.0001	1.574E+007	< 0.0001	Significant
Temp	2.384E+008	< 0.0001	1.198E+008	< 0.0001	
Feed type (B)	7.497E+006	< 0.0001	4.476E+006	< 0.0001	
TB	2.506E+005	< 0.0001	25,257.22	< 0.0001	
T^2^	0.18	0.6733	1.14	0.2991	

*T* temperature term, *B* type of feed supplied.

## Results and discussion

### Effect of various feeds

**Figure 10:**
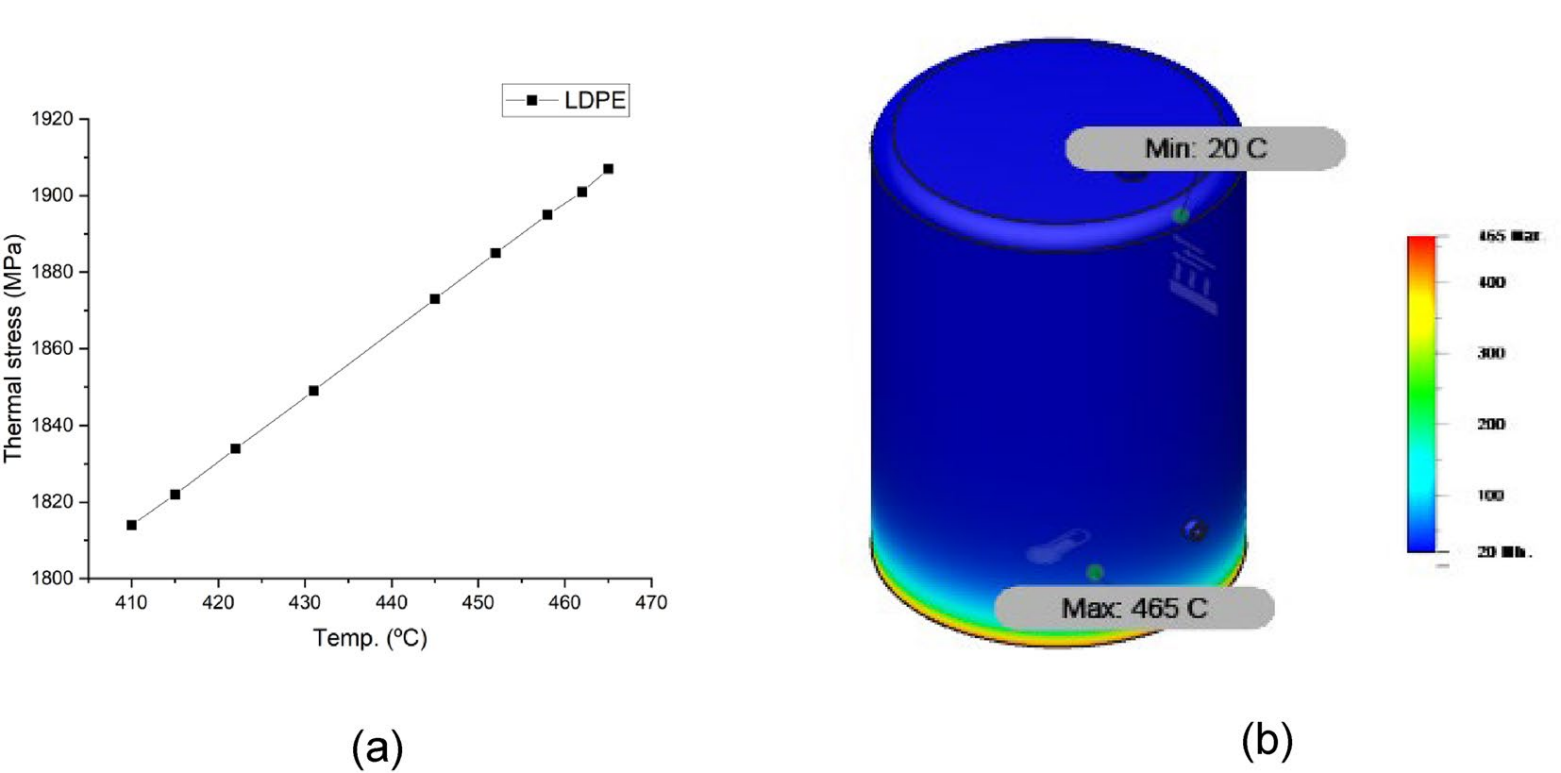
LDPE (**a**) thermal stress curve, (**b**) temperature contour.

**Figure 11:**
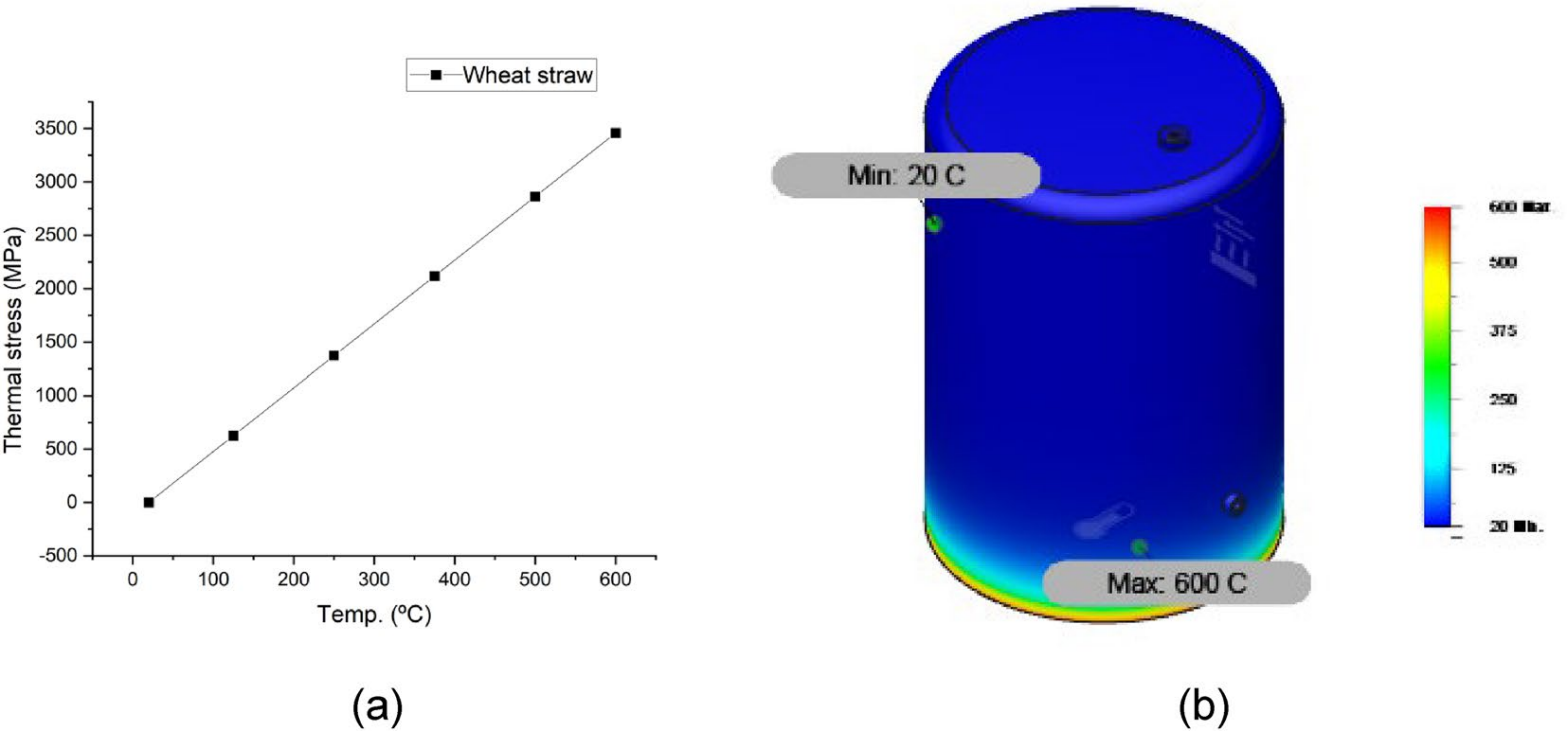
Wheat straw (**a**) thermal stress curve, (**b**) temperature contour.

**Figure 12:**
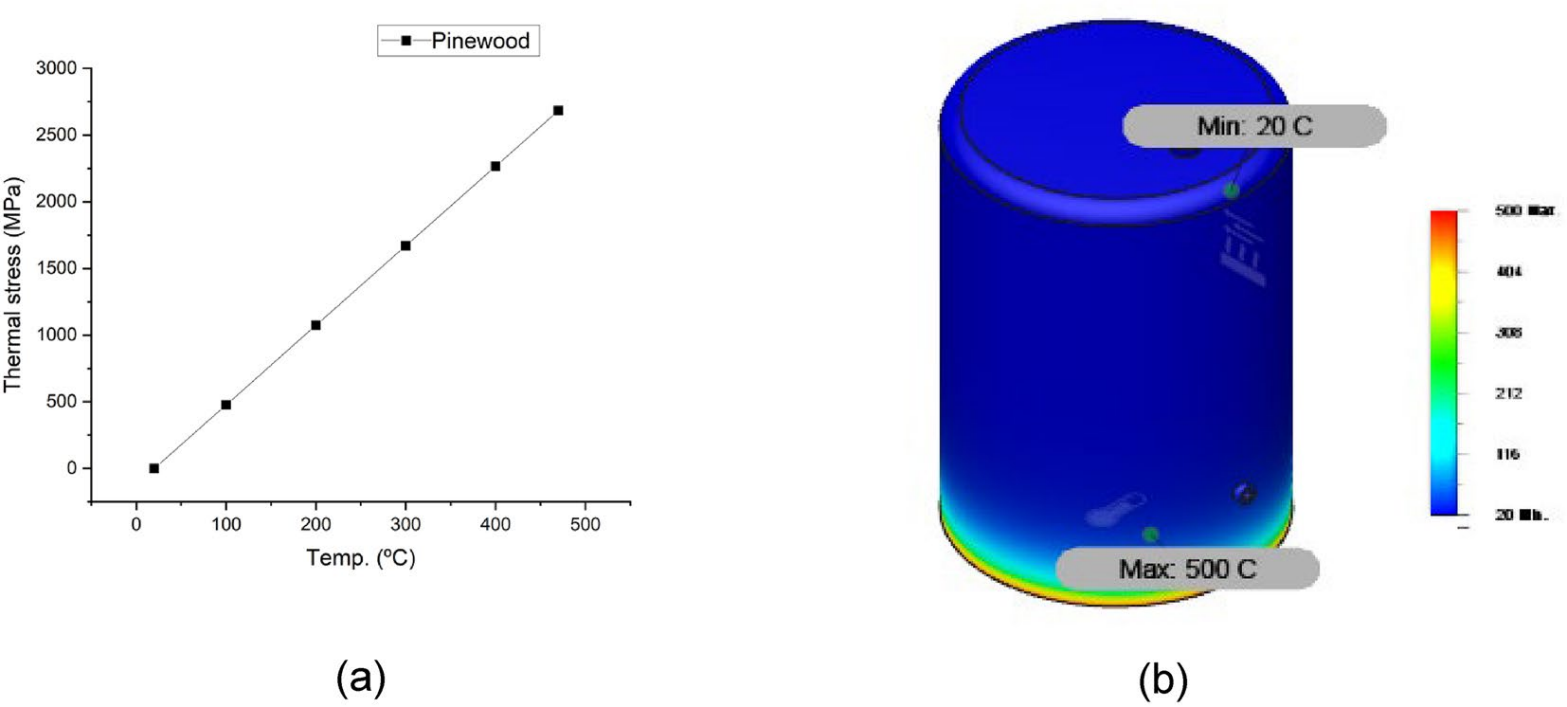
Pinewood (**a**) thermal stress curve, (**b**) temperature contour.

**Figure 13:**
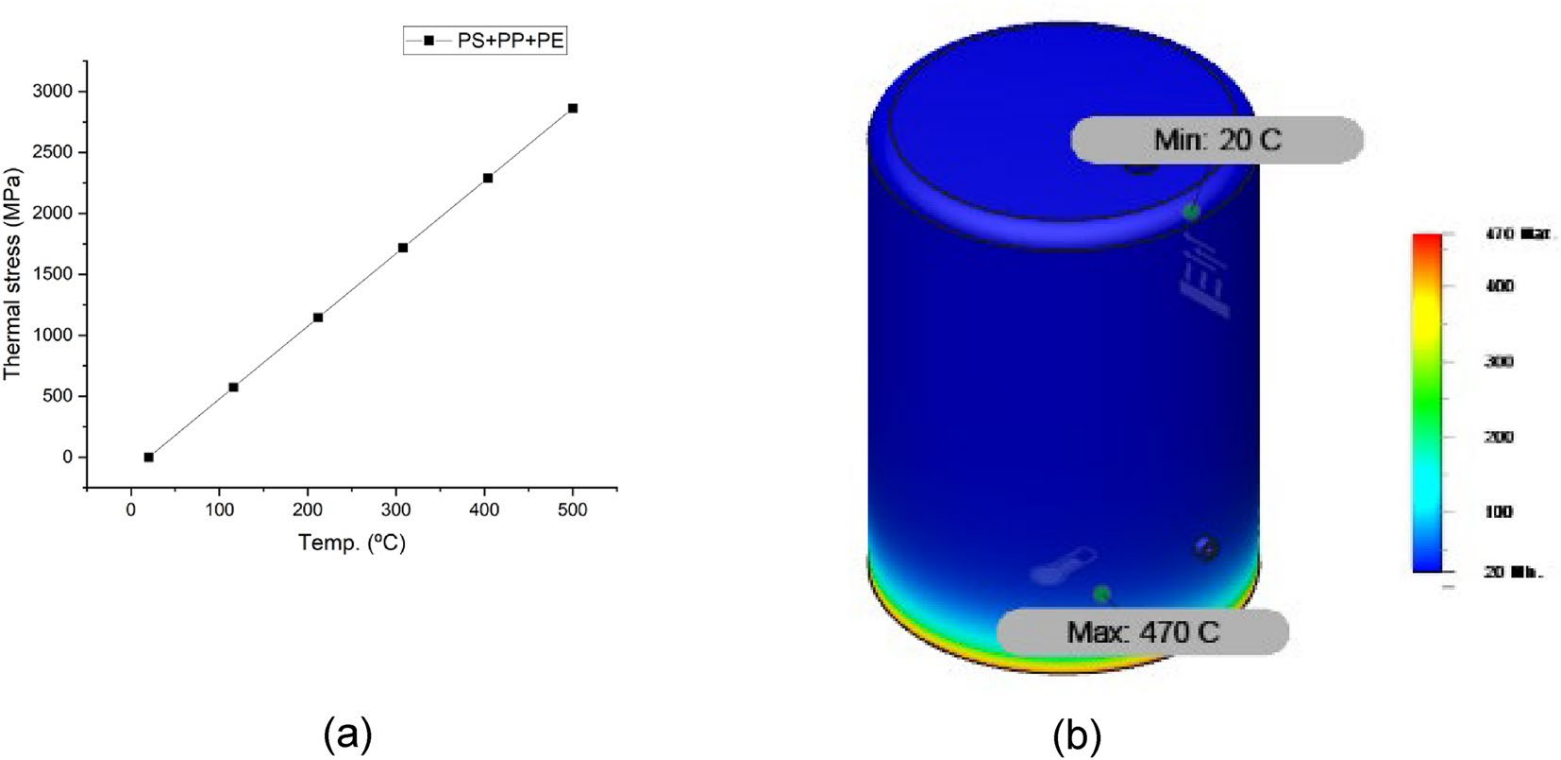
PS + PE + PP (**a**) thermal stress curve, (**b**) temperature contour.

Figures 10, 11, 12 and 13 show the variation of different feeds with pyrolysis temperature. Figure 10 shows LDPE thermal stress curve and temperature contour while Fig. 11 shows the same parameters for wheat straw. Figure 11 depicts the thermal stress and temperature contour of pinewood while Fig. 12 shows the same values for the PS + PE + PP mixture. Based on these results, it was concluded that the thermal stress and strain values increase with increasing temperatures. LDPE got the least thermal stress and strain values, and wheat straw got the highest values.

**Figure 14:**
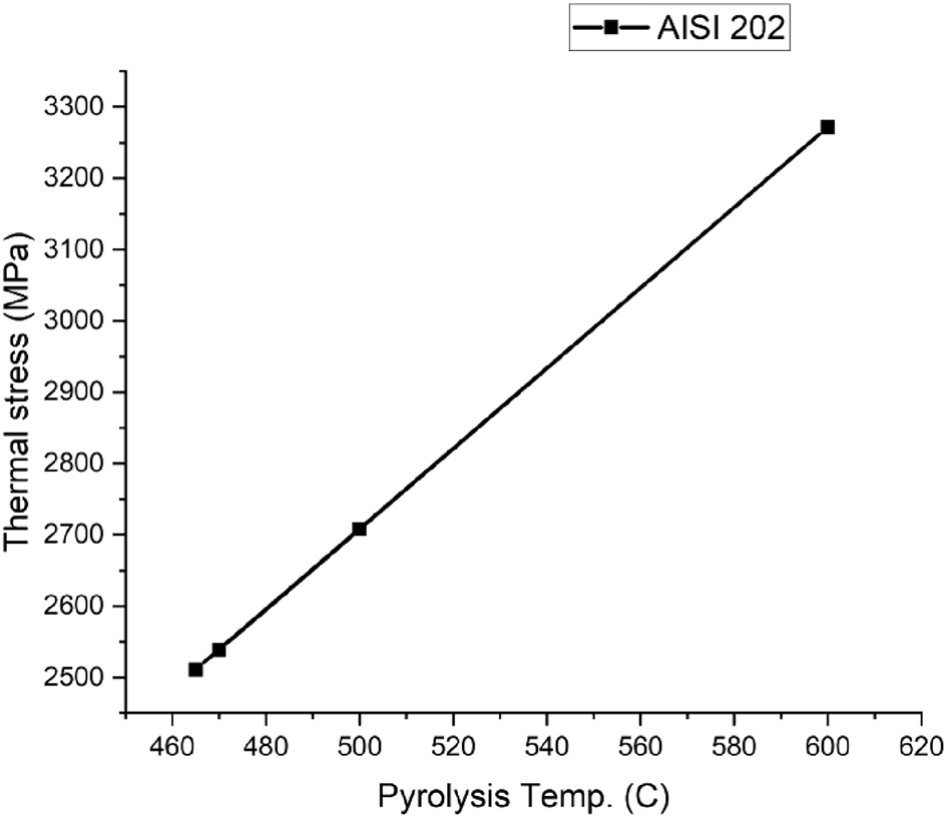
AISI 202 Thermal stress curves.

**Figure 15:**
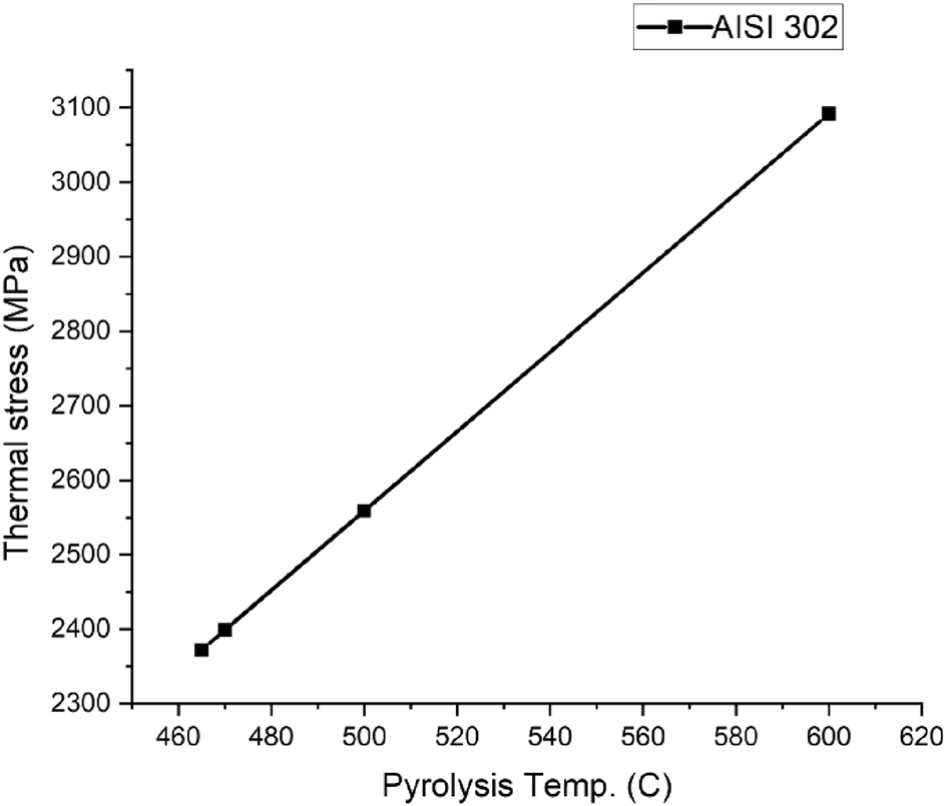
AISI 302 Thermal stress curve.

**Figure 16:**
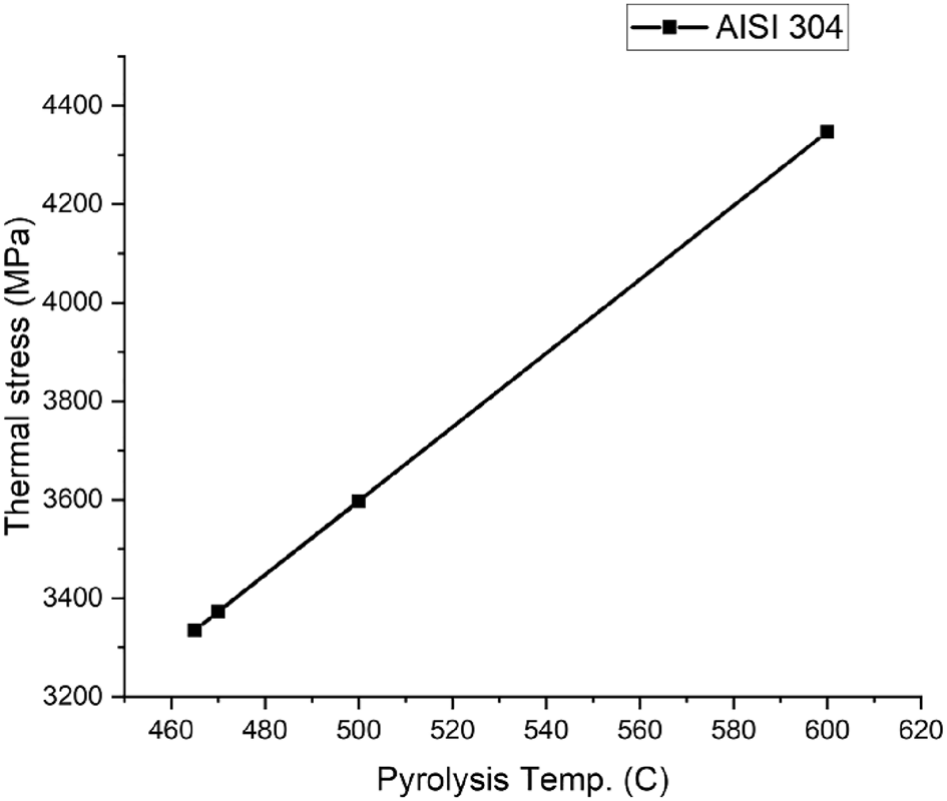
AISI 304 Thermal stress curve.

**Figure 17:**
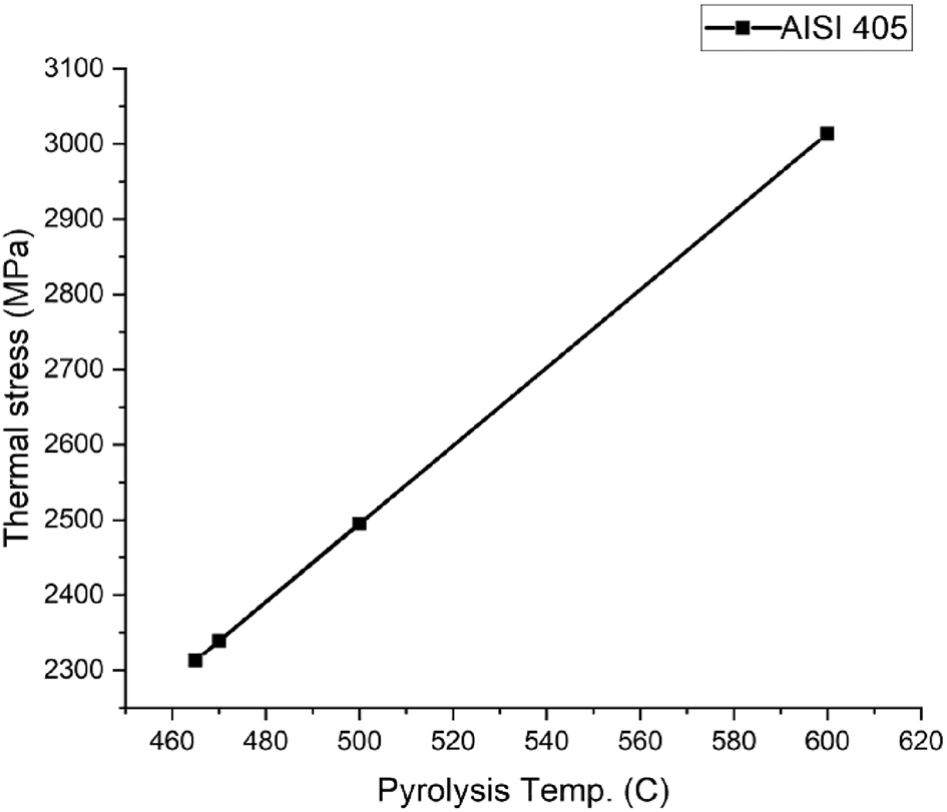
AISI 405 Thermal stress curve.

Figures 14, 15, 16 and 17 show the behavior of different reactor materials with various feeds (having different pyrolysis temperatures). It was observed that Stainless steel AISI 304 could withstand high temperatures and pressures due to the presence of high chromium and nickel content in it.

### Modeling and optimization

The RSM-based models developed in the form of mathematical equations as shown in “Modeling and optimization” section (Eqs. 6–13) were used to predict the model output for different feedstocks and temperatures. The performance of these models and parametric optimization is discussed in the following sections.

The ANOVA of the data also helped in deducing the correlation between independent control factors (temperature and type of feed) and response variables (thermal stress and strain). The algebraic expression developed for thermal stress caused by different types of feeds is shown below in Eqs. (6) to (9):

Feed type: LDPE

6 $$Thermal \,stress = 1120.64 + 1.69T - 1.12E-006\times {T}^{2}.$$

Feed type: WS

7 $$Thermal\, stress = -119.3 + 5.97T - 1.126E-006\times {T}^{2}.$$

Feed type: PWS

8 $$Thermal \,stress = -119.22 + 5.97T - 1.126E-006\times {T}^{2}.$$

Feed type: PPP

9 $$Thermal\, stress = -\,\,119.34 + 5.97T - 1.127E-006\times {T}^{2}.$$

The correlation developed for thermal strain due to different type of feeds are shown below in Eqs. (10) to (13):

Feed type: LDPE

10 $$Thermal \,strain= 1.72E-004 + 2.64E-005T+ 2.59E-011\times {T}^{2}.$$

Feed type: WS

11 $$Thermal\, strain= -\,7.79E-004 + 3.89E-005T + 2.59E-011\times {T}^{2}.$$

Feed type: PWS

12 $$Thermal \,strain = -\,7.78E-004 + 3.89E-005T + 2.59-011\times {T}^{2}.$$

Feed type: PPP

13 $$Thermal\, strain = -\,7.77E-004 + 3.88E-005T + 2.6E-011\times {T}^{2}.$$

#### Thermal stress model

**Figure 18:**
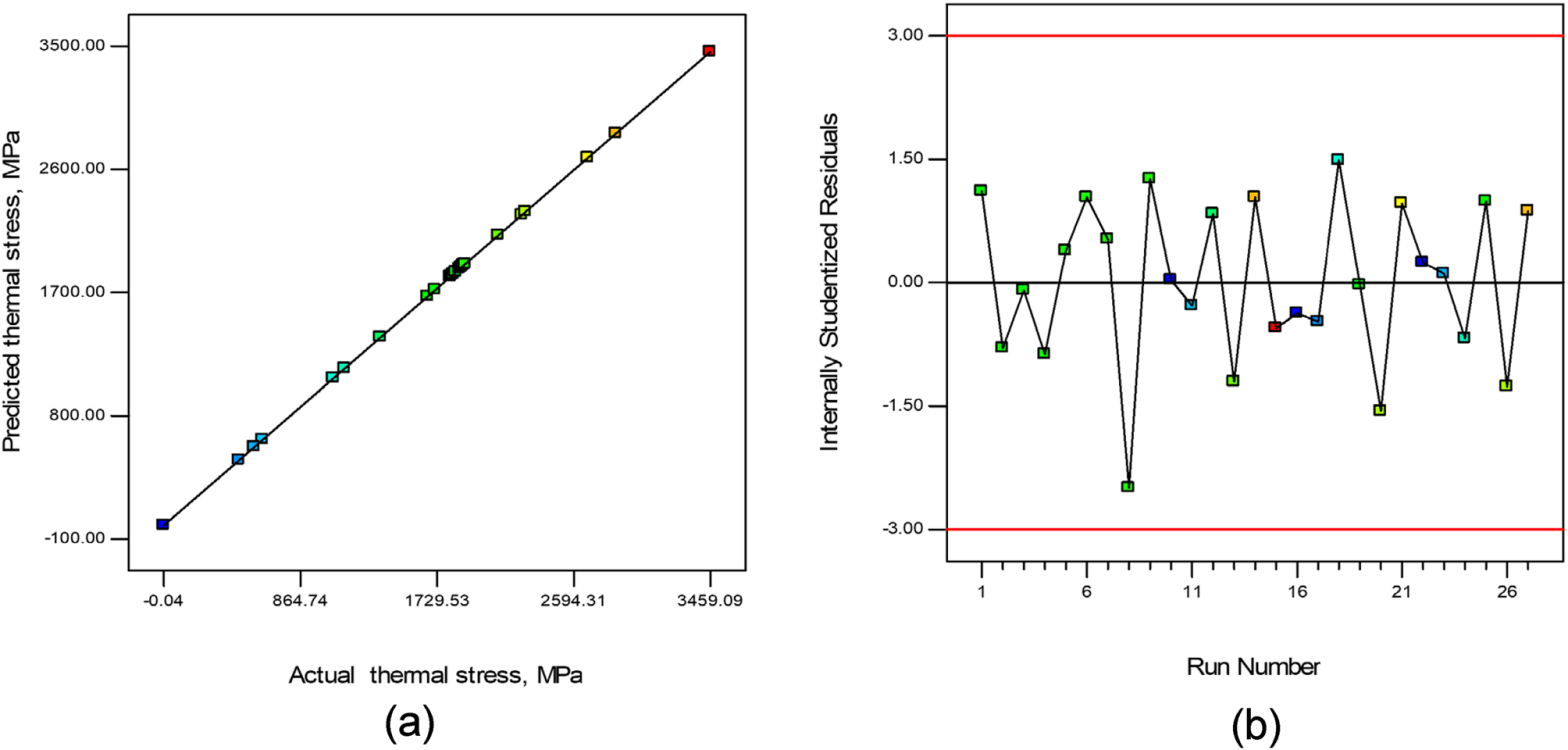
Thermal stress (**a**) Actual vs model predicted values, (**b**) Internally studentized residuals.

Equations (6) to (9) were utilized to predict the outcomes (thermal stress) for all four types of pyrolysis feeds at the whole range of operational parameters. Table 6 shows how the findings were utilized to construct several statistical indices for model assessment. Figure 18a depicts a graphical comparison of observed and forecasted thermal stress developed in the metallic body of the reactor body, whereas Fig. 18b shows a graph of studentized residuals in the model predicted values. The graphical representation of comparison model values and low residuals in Fig. 18a,b shows that the RSM-based developed model was a robust prognostic model. The statistical examination of the results, as shown in Table 6, corroborated this conclusion. The created model produced a low root mean squared error (RMSE) of 0.236, close to unity values of R^2^ as 0.9931, R^2^ (Adjusted) as 0.9902, and R^2^ (Predicted) as 0.9902, indicating an efficient prediction model for thermal stress.

**Table 6:** Statistical evaluation of model’s performance.

	R^2^	R^2^ (Adjusted)	R^2^ (Predicted)	Root mean squared error
Thermal stress model	0.9931	0.9914	0.9902	0.236
Thermal strain model	0.9924	0.9905	0.9898	0.347

#### Thermal strain model

**Figure 19:**
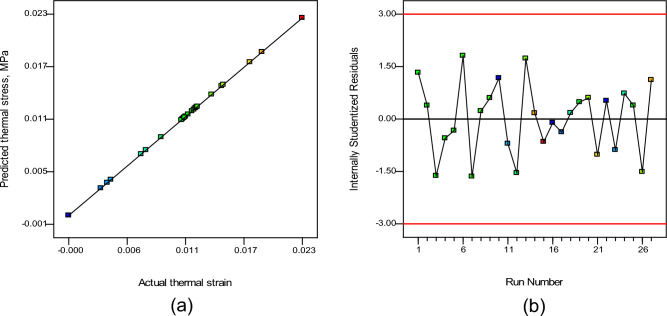
Thermal strain (**a**) actual vs model predicted values, (**b**) internally studentized residuals.

The thermal strain models as expressed with Eqs. (10)–(13), being used to estimate the results for all four types of pyrolysis feeds throughout the whole range of operating parameters. Figure 19a displays a graphical comparison of observed and expected thermal strains in the metallic body of the reactor body, while Fig. 19b provides a graph of studentized residuals in the model predicted values. Figure 19a,b displays a graphical depiction of comparison model values and low residuals, indicating that the RSM-based created model was a robust prognostic model. This model conclusion was supported by a statistical assessment of the findings, as shown in Table 6. The developed model provided a low root mean squared error (RMSE) of 0.347, near to unity values of R^2^ as 0.9924, R^2^ (Adjusted) as 0.9905, and R^2^ (Predicted) as 0.9898, demonstrating an effective thermal stress prediction model.

#### Desirability based optimization

**Figure 20:**
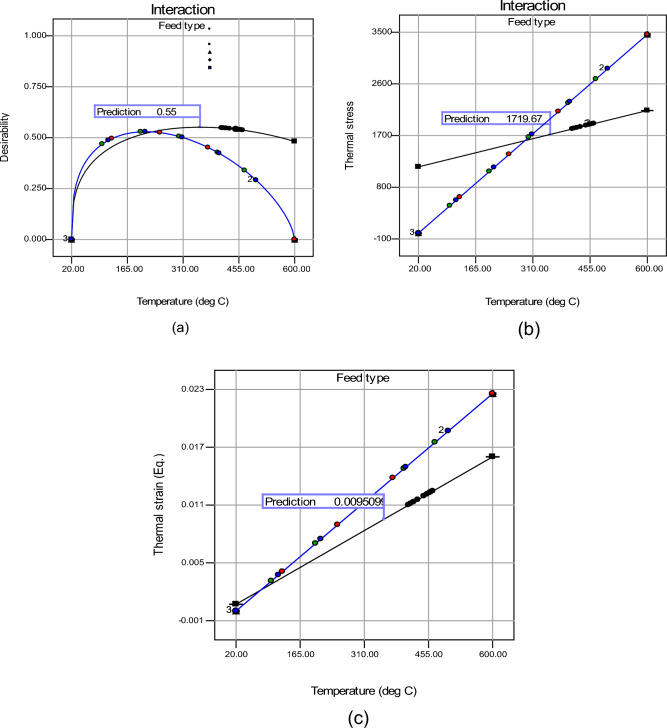
Prediction map of (**a**) Desirability, (**b**) thermal stress, (**c**) thermal strain.

The findings of RSM-based model prediction demonstrated a considerable variation in output response throughout the temperature range and also with the type of feed supplied for pyrolysis. Since the optimal thermal stress–strain paradigm needs a trade-off between control elements, a desirability-based method was used to determine the best operating parameters and feedstock. Table 7 describes the conditions for such an optimization, such as the lower and upper boundaries and the goal provided for each solution. Figure 20a displays a desirability diagram showing the trade-off between different control factors, with a bar graph displaying individual and combined desirability. The option with the highest desire index (0.55) was chosen. The best control factors were revealed at 354 °C with LDPE as the feedstock, as shown in Table 7. At these optimal settings, the best thermal stress and strain responses were 1719.67 MPa (Fig. 20b) and 0.0095 (Fig. 20c), respectively.

**Table 7:** Optimization results.

Optimized input factors		Response variable	
Temperature (°C)	Type of feed	Thermal stress	Thermal strain
354	LDPE	1719.67	0.0095

## Conclusion and future scope

It was concluded that pyrolysis is the panacea for the existing waste management issue. Keeping this vista in mind, a study was conducted on the structural analysis of pyrolysis reactors due to high temperatures and pressures. Fusion 360 was used for the investigation. Four feeds (namely, LDPE, wheat straw, pinewood, and a combination of PS, PE, and PP) and four reactor materials (materials Stainless steel AISI 202, Stainless steel AISI 302, Stainless steel AISI 304, and Stainless steel AISI 202) were considered for the analysis. Based on the study, the following results can be concluded:

- The thermal stress and strain values of the pyrolysis reactor increase with increasing temperatures as seen from the simulation results.
- Amongst the 4 feeds studied in this paper, LDPE comes out to be the most effective feed based on low pyrolysis temperature and low-stress values. Low-stress values favor the process as the reactor deformation is less and therefore, it can withstand more heat.
- Out of the different grades of stainless steel discussed in this paper, AISI 304 has the best ability to withstand high temperatures owing to its high chromium content of 18–20% and nickel content of 8–11%. Moreover, it can withstand the highest stress of all.
- RSM was successfully employed for the development of a prognostic efficiency with high efficiency having R^2^ (0.9924–0.9931) and low RMSE as (0.236 to 0.347).
- Desirability-based optimization revealed the optimized pyrolysis temperature of 354 °C with LDPE as the feedstock. At these optimal settings, the best thermal stress and strain response were 1719.67 MPa and 0.0095, respectively.

This paper gives an insight into the structural and thermal analysis, and Response Surface Methodology (RSM) of the pyrolysis reactor by considering various feeds and reactor materials. Simulations help solve real-world problems efficiently and safely while taking into account all the necessary conditions required for the process. The reactor considered here is optimized to work at 354 °C, having thermal stress and strain response at 1719.67 MPa and 0.0095, respectively.

### Future scope

Waste to energy is one method that can implanted to address the energy demand of a nation. In this regard, pyrolysis is one of the methods that can be adopted successfully. In this regard, optimisation of the pyrolysis temperature for different feedstocks like municipal waste, medical waste and agricultural residue can attribute to efficient conversion of waste to energy.

## Data Availability

The datasets used and/or analysed during the current study available from the corresponding author on reasonable request.

## Abbreviations

AISI: American Iron and Steel Institute
ANOVA: Analysis of variance
BET: Brunauer–Emmett–Teller
FEA: Finite element analysis
LDPE: Low-density polyethylene
PE: Polyethylene
PP: Polypropylene
PPP: PS + PP + PE
PS: Polystyrene
PWS: Pinewood
RSM: Response surface methodology
R^2^: Coefficient of determination
WS: Wheat straw
